# The Development of a Team-Based, Hybrid Inter-university Graduate Certificate Program Focused on Maternal Child Health Professionals

**DOI:** 10.1007/s10995-022-03455-w

**Published:** 2022-07-29

**Authors:** K. Tollestrup, T. Thomas, N. Stone, S. Chambers, P. Sedillo, F. Perry, S. Forster-Cox

**Affiliations:** 1 College of Population Health, University of New Mexico, MSC 09-3070, Albuquerque, NM 87131-0001 USA; 2 Department of Public Health Sciences, New Mexico State University, P.O. Box 30001, MSC 3HLS, Las Cruces, NM 87131-0001 USA

**Keywords:** Maternal and child health academic training, Hybrid learning, Graduate education

## Abstract

**Introduction:**

Pregnancy, childbirth, and child well-being are identified by Healthy People 2030 as priority topics for improving the health of all Americans. New Mexico is the fifth largest state geographically with most of the state’s 33 counties considered rural or frontier. Accessing health care services is challenging in this resource-poor environment. The need to provide maternal and child health (MCH) education in the state was the impetus for developing a graduate certificate in maternal and child public health.

**Methods:**

The hybrid MCH graduate certificate engaged professionals in formal training that included a public health approach to addressing MCH issues in the state’s diverse communities. Grant funds paid for the tuition, books and travel for students providing an opportunity to individuals who otherwise could not have pursued graduate education and professional development.

**Results:**

Over a 4-year period, two cohorts were recruited, educated, and evaluated. The evaluations reflected an increase in competency knowledge scores for all students.

**Discussion:**

This model of MCH education was successful at delivering public health graduate education to MCH practitioners and increasing their knowledge and skills. Listening to students and communities as to what their MCH public health needs are and responding with a flexible educational model provided individuals with information and tools that could be used to improve maternal and child health and reduce health disparities in rural, tribal, and underserved communities.

## Significance Statement

*What is already known on this subject?* Public health training for maternal and child health practitioners, located in rural, tribal, border and underserved areas, is critical for improving the health of the diverse communities they serve. Traditional graduate level training is challenging for professionals in rural areas. This model provides a practical method to serve MCH training needs in rural areas.

*What this study adds?* We provide a description of a successful hybrid public health certificate for maternal child health practitioners working in rural, tribal, and underserved communities. Lessons learned and challenges to this type of educational training program are also described.

## Introduction and purpose

Pregnancy, childbirth, and child well-being are identified by Healthy People 2030 as priority topics for improving the health of all Americans (Office of Disease Prevention and Health Promotion, 2021). There are many challenges in addressing maternal and child health (MCH) in the United States. Particular barriers to improving MCH vary according to demographics, geography, access to health care, culture, and social determinants of health. Therefore, training programs focusing on improving MCH outcomes must be tailored to specific communities and ultimately designed and implemented by those who know the needs of the communities served. Providing MCH training to individuals from and working in these diverse and often underrepresented communities is one strategy to improving MCH outcomes (Ahn et al., 2020).

New Mexico (NM) is a state with tremendous needs for MCH trained professionals. According to the Annie E Casey Kids Count data, New Mexico has ranked 50th among US states for overall child well-being since 2013 and is 49th as of 2021 (New Mexico Voices for Children, 2022; Annie & Casey, 2020). The NM poverty rate is 18.2% and is second in the nation for child food insecurity with 1 in 4 children struggling with hunger (Feeding America, 2020; US Census Bureau, 2020). Due to the rural and frontier nature of the state, health care and public health access is challenging. This is especially true for the more sparsely populated rural areas of the state where it difficult to hire and maintain full-time clinicians and specialists. Other barriers to access include a general provider shortage, lack of affordable insurance, and having to travel long distances for routine and specialty care. Thirty-two of NM’s thirty-three counties are classified as “health professional shortage areas” (Maternal Child Health Services Title V Block Grant, 2019). These realities, along with inadequate MCH funding and the challenges of addressing health inequities in a multicultural state, speak to the need for practitioners who can address the needs of children, women, and families.

This article discusses the development of a hybrid MCH graduate certificate in public health in NM. The goal of the MCH graduate certificate was to create a dynamic and innovative public health training curriculum for MCH professionals that would improve their public health knowledge, enabling them to address health disparities in rural, tribal, and underserved communities. The pre-pandemic hybrid design, with in-person sessions once a semester, and an online learning platform allowed people in rural, tribal, and border areas to seek new knowledge and professional development. The MCH graduate certificate was a collaboration between academic institutions and public health partners. It engaged professionals in formal training that included a public health approach to addressing MCH issues in the state’s diverse communities. Moreover, the certificate strengthened students’ leadership skills as MCH professionals serving in these unique areas, through collaborative projects and shared expertise.

## Description of MCH Graduate Certificate

This MCH graduate certificate was established as a culturally competent MCH training program through the New Mexico MCH Public Health Training Institute (MCH-TI) and was funded by the Health Resources and Services Administration. The 12-credit MCH graduate certificate began in 2014 and was designed by an interdisciplinary team of public health professionals. The interdisciplinary team included faculty and staff from University of New Mexico (UNM) College of Population Health (COPH), UNM Center for Rural and Behavioral Health in the Department of Psychiatry (CRCBH) and New Mexico State University Department of Public Health Sciences (NMSU). This group had expertise in epidemiology, community health education, leadership and management, behavioral health, and public health practice. The goals of the certificate were to: (1) to create a dynamic and innovative public health training curriculum for MCH professionals; (2) to engage MCH professionals in formal training including a public health approach to addressing MCH issues in rural, tribal and underserved communities; and (3) strengthen the leadership skills of MCH professionals serving these communities through collaborative projects and shared expertise.

The curriculum was developed to be a hybrid delivery approach (face-to-face and online classes) and completed in 2 years, with all courses designed to be transferable for credit into a Master of Public Health (MPH) or related graduate programs. The 12 credits of coursework were delivered using a combination of face-to-face workshops, online courses, telehealth seminars and webinars, and consisted of four graduate courses: Essentials of Public Health for MCH, Essentials of Epidemiology for MCH, MCH Public Health Planning, and MCH Public Health Leadership and Management. Additionally, the curriculum included a capstone experience, which included two learning activities. The first was a written capstone paper focusing on a specific MCH issue relevant to the trainees’ work experience and a broader issue within the Southwest. The second was an individual poster presentation made at the statewide New Mexico Public Health Association annual conference. The first cohort began the certificate coursework in the spring semester in January 2015 and completed the certificate at the end of the fall semester in December 2016. The second cohort began in the spring semester in January 2017 and completed the certificate at the end of the fall semester in December 2018. Funding from the grant was used to support the trainees including tuition, travel and conference expenses.

Based on trainee feedback, the curriculum was adjusted to better serve the needs of participants. Throughout the funding period, the course competencies and learning objectives remained the same (Table 1). The major change in the curriculum was course timing. Students in the first cohort completed their 12 credits with one course per semester including two summer sessions. Because summer sessions proved difficult for trainees, the curriculum was modified for the second cohort so that courses were only offered in the fall and spring semesters.

**Table 1:** MCH Competencies used in each certificate course

MCH graduate certificate course	Competencies
Essentials of Public Health for MCH	The major domestic and international causes of mortality and morbidity within MCH populations including differences between the United States and other developed and less developed countries
	The determinants of health and illness, and concomitant theories including biological, behavioral and socio-cultural influences such as racism, sexism, and economic disparity, as well as protective factors
	The principles and theories of population-based health promotion at the individual, family and community levels
	Critically analyze inequities in health status based on race/ethnicity, socioeconomic position, and gender
	Recognize different strengths, needs, values, and practices of diverse cultural, racial, ethnic, and socioeconomic groups and determine how these factors affect health status, health behaviors, and program design
	The philosophical concepts and rationale underlying the delivery of family-centered, comprehensive, community-based, and culturally competent MCH and public health services and programs, including recognition of community assets
Essentials of Epidemiology for MCH	Epidemiological concepts and descriptive epidemiology
	The use of data to illuminate ethical, political, scientific, economic, and overall public health issues
	Strengths and limitations of qualitative and quantitative methods
	Data collection strategies and their strengths and limitations, including surveys, focus groups, and record-based information
	Prepare and interpret data from vital statistics, censuses, surveys, service utilization, and other relevant reports on the health of MCH populations and have the ability to detect meaningful inferences from data and the translation of data into information
	Ability to conceptualize and appropriately use data and statistical/epidemiological methods for problem and asset identification, assessment, program planning, implementation, and evaluation
	Evaluate the integrity and comparability of data and identify existing gaps
MCH Public Health Leadership and Management	Organizational and management theories and practices
	Appropriate use of networking, advocacy, negotiation, and conflict resolution skills
	Resolution of internal employee and/or organizational conflicts through knowledge of applicable management techniques
	The principles and issues involved in the ethical and sensitive conduct of practice within MCH populations, and in the organization and delivery of public health services within communities
	Ethical conduct in practice, program management
MCH Public Health Planning	How to apply knowledge of demographic, health, familial, socio-cultural, environmental, and community factors to the design of MCH programs and services
	Effective written and oral communication skills, including accurate and effective preparation and presentation of reports to agency boards, administrative organizations, legislative bodies, consumers, and/or the media using demographic, statistical, programmatic, and scientific information
	Recognize different strengths, needs, values, and practices of diverse cultural, racial, ethnic, and socioeconomic groups and determine how these factors affect health status, health behaviors, and program design
	The theories and principles of community organization, change, and development

The grant paid the tuition and books for the students, which was approximately $2100/semester. The grant also paid for the students to travel from their home communities to Albuquerque for the start of the semester face-to-face meetings and hotel costs, plus registration to the annual meeting of NMPHA.

### Recruitment

Marketing activities targeted all MCH agencies and other groups who serve MCH populations in the state. Information about the certificate was sent to several large electronic mailing lists including those from New Mexico Public Health Association (NMPHA), the COPH and the NMSU Master of Public Health Program. Personal e-mails were also sent to MCH colleagues in New Mexico, and we talked in-person to individuals who we knew were influential in the MCH arena in the state. Flyers were distributed at all MPH recruitment fairs, including annual public health conferences, ‘Public Health Day” at the state legislature local educational fairs and various email list-servs. Oral presentations about the certificate were regularly accepted at the NMPHA annual meetings.

### Curriculum

The four core courses for the 2-year certificate included Essentials of Public Health for MCH, MCH Public Health Leadership and Management, Essentials of Epidemiology for MCH, and MCH Public Health Planning. Essentials of Public Health for MCH focused on understanding the critical role of public health today and taught students how to make a public health impact through their work. MCH Public Health Leadership and Management provided students with a working knowledge of healthcare management and application of leadership, ethics, and management principles to the MCH work environment. Essentials of Epidemiology for MCH provided students with basic epidemiologic knowledge and skills used to analyze and interpret disease occurrence in populations. MCH Public Health Planning focused on public health issues related to MCH and provided students with knowledge, practical skills, and tools to conduct health assessments and write a basic intervention funding proposal.

For each course, competencies were reviewed from the MCH Leadership Competencies (Department of Health and Human Services, 2018) and the Core Competencies for Public Health Professionals (Council on Linkages between Academia and Public Health Practices, 2010). These competencies were designed as a conceptual framework for training programs to develop and evaluate curricula and identify knowledge and skill areas required of MCH leaders. We selected four to six specific competencies for each course to guide our development of the course materials.

### Evaluation

The MCH graduate certificate curriculum was monitored and assessed throughout the 5-year grant period. The UNM Institutional Review Board reviewed the certificate evaluation methods and granted the project exempt status. All students gave informed consent prior to inclusion in the study.

Our evaluation approach included both process measures and a quantitative assessment of changes in knowledge and skills of the students. The process measures were important in identifying issues and problems in implementing the graduate certificate and allowed us to solve the issues since they were identified early. At the end of each course, a Plus/Delta formative evaluation was used during a debriefing session with the certificate participants. This formative approach is designed to gather information quickly and easily on what worked, what did not work and suggestions for improvements and revisions (Fanning & Gaba, 2007). Qualitative information was also gathered from students on general course evaluation measures and included examples of how the students were applying what they learned to their job. Additional interviews (individual and group) were held with students at the end of the first and second years of the program. Topics covered career goals, graduate school plans, and ways in which the program was benefitting the student’s interactions with clients, co-workers, and organizations.

Pre- and post-assessments were completed for each course and measured the students’ own assessment of their degree of knowledge about the course-specific competencies listed in Table 1. Students used a four-point Likert scale to describe the degree they were knowledgeable about the specific competency: 1 = none, 2 = aware, 3 = knowledgeable and 4 = proficient. An overall knowledge score for a course for each student was calculated as an average of the student’s knowledge scores for each course-specific competency. Pre- and post-course overall knowledge scores were calculated for each student for each course. The Wilcoxon signed rank test was used to identify statistically significant changes between the pre- and post-knowledge scores for each course.

## Results

Recruitment for the two cohorts was successful at creating a diverse pool of applicants that reflected the diversity of the state (Table 2). Of the 41 applicants who applied for the certificate, 34% were Hispanic and 15% were Native American. The applicants were employed by a variety of institutions in the state including health care settings (hospitals and community clinics), tribal agencies or the Indian Health Service, and educational institutions (universities and public schools). Most applicants were health educators or in a teaching position. A total of 27 of the applicants were accepted. Of these, 13 completed the certificate and reflected a similar diversity in race/ethnicity, employer and employment position (Table 2). Reasons for not completing the certificate were varied. They included not having enough time to devote to the coursework, not having adequate and reliable internet access, changing and increased family obligations, and moving to a new job position.

**Table 2:** Characteristics of applicants who applied for the MCH graduate certificate and graduates who earned the certificate

Characteristic		All applicants (n = 41)	All graduates (n = 13)
Gender	Female	3	13
	Male	38	0
Race/ethnicity	Non-Hispanic white	12	4
	Hispanic	14	5
	Native American	6	4
	African American	1	0
	Unknown	8	0
Employer	State health or another department	6	2
	Non-profit public health or health agency	6	3
	Tribal agency or Indian Health Service	8	4
	Public university or school	10	2
	Healthcare system or clinic	10	2
	Self-employed	1	0
Position	Health educator or health promotion specialist	17	8
	Supervisor or program manager	14	3
	Nurse, midwife, or doula	9	2
	Student	1	0

Every student demonstrated an increase in their course competency knowledge score for each of the four courses. Post-course overall knowledge scores were significantly higher than pre-course overall knowledge. The largest increase in the course overall knowledge score was for Essentials of Epidemiology for MCH where students’ scores increased on average 1.14 points. On average at the end of each course, students felt they were knowledgeable to proficient in the competencies covered by the specific course (Table 3).

**Table 3:** Overall average competency knowledge score for students completing each MCH graduate certificate course

Course	Sample	Mean Overall Knowledge Score Pre-assessment	Mean Overall Knowledge Score Post-assessment	p-value
Essentials of Public Health for MCH	6	2.42	3.08	< 0.05
Essentials of Epidemiology for MCH	9	1.81	2.98	< 0.05
MCH Public Health Leadership and Management	10	2.45	3.32	< 0.05
MCH Public Health Planning	4	2.72	3.19	^1^

^1^Sample size too small to calculate p-value

Follow-up was completed for the two cohorts to determine whether they continued their graduate education which was one main goal of the training program. A total of 9 (69%) of the graduates continued taking additional courses, participated in certificates (Public Health Training Certificate for American Indian Health Professionals, Community Health Worker Certification), became fellows or scholars (Johns Hopkins University Center for American Indians Scholar, Data Scientist Immersive Fellow), or entered nursing school or master’s programs in public health, health education, public administration, or Spanish.

Information from the Plus/Delta formative evaluation for each course was used to revise and refine the course for the next cohort. For example, the two summer session courses were dropped for the second cohort based on feedback from the first cohort. Students in the first cohort suggested using weekly modules for the online course content and assignments, and the structure of the Essentials of Epidemiology for MCH course was revised to reflect this. The second cohort stated that the 2-year curriculum was quite long given the ambitions and commitments of the students.

Students were recruited from the MCH workforce, and most had not engaged in graduate level education. Students described their newfound confidence in pursuing higher education. Quotes include, “I feel like I use this as a stepping-stone. Ultimately this isn’t a master’s but whatever program we do decide to go to, we use the certificate.” Another student added, “This certificate class has made me less anxious about applying to graduate school and knowing I can do it.”

Students also gained valuable skills that they could use in their work. One student shared, “I feel like I have some new tools that will allow me to better serve my clients. I also broadened my perspectives of how to collaborate to make change and got a better overall picture of the various ways that MCH programs overlap and interface.” Another said, “I will be able to understand how policy will impact communities in a way that I have not understood or even thought about in the past. I will not only have more knowledge about related issues, but also know where to find information to understand concepts at a deeper level.”

Students also felt that funding was beneficial in completing the certificate. One student said, “Having paid registration, travel and food was really beneficial.”

## Discussion

The MCH graduate certificate created a dynamic and innovative public health training curriculum for MCH professionals who work to improve the health and reduce health disparities in NM’s rural, tribal, and underserved communities. The development, implementation and evaluation of the program provide an important MCH resource for NM and other states. Offering the certificate courses online with one in-person session per semester was valuable as students could remain in their communities and at their jobs while obtaining new knowledge and skills and gave them the opportunity to bond with each other during the in-person sessions (Jowsey et al., 2020). Providing resources and funding to pay for tuition, books, travel, and other costs was crucial for most students and allowed them to complete the courses and earn the certificate.

### Lessons learned

The following are some of the important lessons learned to provide and improve MCH education in rural, tribal, and underserved communities. Having courses that were designed to allow for immediate implementation of knowledge in current workplaces was valuable. Utilizing a hybrid educational format, with much of the courses delivered online allowed for wider participation in the certificate. While using an online format required the students to gain new technology skills, it ensured reach into underserved communities. The novel format also encouraged instructors to revise and enhance their historical course content and curriculum to include interactive online discussions, video sources and other media. Essential in-person sessions were kept to a minimum and where important networking and continued collaboration happened among students, mentors and instructors.

A bonus for many students was the enhanced confidence they realized, having successfully completed the certificate. Some students had previously thought they could not accomplish graduate work, when in fact, they performed very well. This enhanced confidence was likely a factor in the decision of over two-thirds of the graduates to pursue additional training and advanced studies, including master’s degrees.

Many students had to overcome barriers to internet access. Much of NM is rural and lacks full access to internet connectivity, yet students were creative and found ways to access the internet including driving to local library parking lots to obtain an internet connection to upload assignments.

Recruitment and retention were another challenge experienced over the years. While the team used its extensive networks throughout the state, recruiting potential students, especially from rural, tribal, and undeserved areas, to apply for the certificate was difficult. Obtaining support from one’s administration, especially to attend the 2-day start of the semester, face-to-face meeting, proved difficult for some. For many who dropped out, the time commitment of the graduate classes, when melded with work, family, and community responsibilities, was too difficult to balance.

Funding for tuition costs and student activities was also an important component of this program. In the interviews and informally during the course of the certificate program, students commented that having tuition covered was one reason for applying to the program. Employed practicing MCH professionals usually do not make high salaries. Thus, the opportunity to have a certificate program that paid tuition was an incentive for applying to and completing the program.

The creation of this certificate led to the development and approval by the UNM Faculty Senate of a new online MCH minor and MCH certificate for undergraduate students. Plans are underway to submit a proposal for a MCH certificate for graduate students. This will be the first MCH minor or certificate offered at an academic institution in NM. The ability to offer the minor and certificate online will allow students and others living in rural, tribal, and underserved areas of NM to obtain pertinent MCH skill sets and knowledge.

A third cohort occurred in the last year of funding, and the certificate was re-designed to be completed in 1 versus 2 years. This change was based on feedback from the students who suggested that a shorter time frame would be more practical and acceptable. The required course credits were dropped from 12 to nine or ten credits, depending upon whether students completed an extra scholarly paper. The epidemiology and public health planning courses were shortened into eight versus 16 weeks.

## Conclusions

The value of the online public health certificate in MCH is significant since it provides a means for increased training and professional development for MCH practitioners in the state. Moving forward to maintain and grow the MCH certificate will require some targeted marketing across NM’s public health and healthcare communities. A necessary emphasis will be the skills obtained, the ability to apply new knowledge immediately at the work site while completing the certificate and that it can serve as a steppingstone to a graduate degree. The ability of other communities and educational institutions to build and deliver a MCH graduate certificate is feasible. Listening to students and communities as to what their MCH public health needs are, and responding with a flexible educational model, will improve the health and reduce MCH disparities in rural, tribal, and underserved communities.

## Data Availability

Curriculum materials are available upon request.
